# Persistent ^131^I uptake in the thyroglossal duct after thyroid ablation: A case report and literature review

**DOI:** 10.1097/MD.0000000000048268

**Published:** 2026-04-24

**Authors:** Jie Qin, Ping Yu, Yu Long Zeng, Jing Ze Li, Xing yu Mu, Zu Guo Li, Wei Xia Chong, Lei Zhang, Bi yun Mo, Wei Fu

**Affiliations:** a Department of Nuclear Medicine, Affiliated Hospital of Guilin Medical College, Guilin, China; b Guangxi Key Laboratory of Drug Discovery and Optimization, School of Pharmacy, Guilin Medical University, Guilin, China; c Shanghai Key Laboratory of Molecular Imaging, Shanghai University of Medicine and Health Sciences, Shanghai, China; d Department of Endocrinology Department, Affiliated Hospital of Guilin Medical College, Guilin, China.

**Keywords:** ^131^I whole-body scan, differentiated thyroid carcinoma, SPECT/CT, thyroglobulin, thyroglossal duct remnant

## Abstract

**Rationale:**

Persistent midline radioiodine uptake after thyroid remnant ablation can mimic metastatic disease and lead to unnecessary re-treatment. We report a case of persistent iodine-131 (^131^I) uptake in the thyroglossal duct region with undetectable thyroglobulin (Tg).

**Patient concerns:**

A 45-year-old woman was found to have thyroid nodules on routine ultrasound, and further evaluation supported papillary thyroid carcinoma.

**Diagnoses:**

After total thyroidectomy and central neck dissection, post-ablation and follow-up ^131^I imaging demonstrated persistent focal uptake in the midline neck. Single photon emission computed tomography, computed tomography (SPECT/CT) localized the activity to the thyroglossal duct tract, while repeated serum Tg remained undetectable and no structural lesion was identified, supporting a benign thyroglossal duct remnant.

**Interventions:**

The patient underwent total thyroidectomy with central neck dissection, followed by 100 mCi ^131^I remnant ablation after thyroid hormone withdrawal. She then received levothyroxine for thyroid-stimulating hormone suppression. Six months later, levothyroxine was withdrawn again for 4 weeks, and a low-dose diagnostic ^131^I scan (3 mCi) with SPECT/CT was performed.

**Outcomes:**

Diagnostic ^131^I imaging at 6 months again demonstrated persistent uptake at the same thyroglossal duct site, with no uptake in the thyroid bed, undetectable Tg, and no structural evidence of recurrent disease. The patient was managed conservatively with thyroid-stimulating hormone suppression and periodic biochemical and ultrasound follow-up.

**Lessons:**

For isolated midline ^131^I uptake after ablation, SPECT/CT localization along the thyroglossal duct tract combined with undetectable Tg supports a benign thyroglossal duct remnant and favors observation with structured follow-up over empiric repeat ablation.

## 1. Introduction

### 1.1. Patient information

A 45-year-old female with no significant past medical history was found incidentally to have a thyroid nodule during a routine health examination. Neck ultrasound revealed 2 solid hypoechoic nodules in the left thyroid lobe (largest approximately 1.0 cm), classified as TI-RADS 4C (In this report, the TI-RADS 4C category refers to the Chinese Thyroid Imaging Reporting and Data System (C-TIRADS), in which category 4 is subdivided into 4A, 4B, and 4C, with 4C denoting a highly suspicious nodule.) according to the Chinese Thyroid Imaging Reporting and Data System (C-TIRADS), indicating a highly suspicious nodule with a high probability of malignancy, along with suspicious lymph nodes in the left prelaryngeal (Delphian) and central neck compartments. The patient had no symptoms of thyroid dysfunction or compression. Ultrasound-guided fine-needle aspiration cytology of the dominant thyroid nodule was performed, revealing clusters of follicular cells with enlarged pale nuclei and papillary structures, consistent with papillary thyroid carcinoma. The patient was informed of the diagnosis and elected to undergo definitive surgical management.

One month after the initial diagnosis, the patient underwent total thyroidectomy with bilateral central neck lymph node dissection at our hospital. Intraoperatively, both recurrent laryngeal nerves were preserved. Pathology confirmed 2 foci of classic papillary thyroid carcinoma in the left lobe (sizes 10 and 5 mm) without extrathyroidal extension or angioinvasion. Metastatic carcinoma was present in 1 of 1 excised Delphian (prelaryngeal) lymph node and 2 of 9 left central compartment lymph nodes (right central nodes 0/7). Background thyroid tissue showed Hashimoto’s thyroiditis. The final pathological staging was pT1bN1aM0 (AJCC 8th edition), consistent with intermediate-risk of recurrence. The patient’s recovery from surgery was uneventful, and she was referred for postoperative radioactive iodine ablation in light of the lymph node metastases (an indication for ^131^I ablation in intermediate-risk differentiated thyroid carcinoma [DTC]). She was advised to hold levothyroxine to elevate thyroid-stimulating hormone (TSH) levels and adhere to a low-iodine diet for several weeks before ablation.

**Figure 1.**
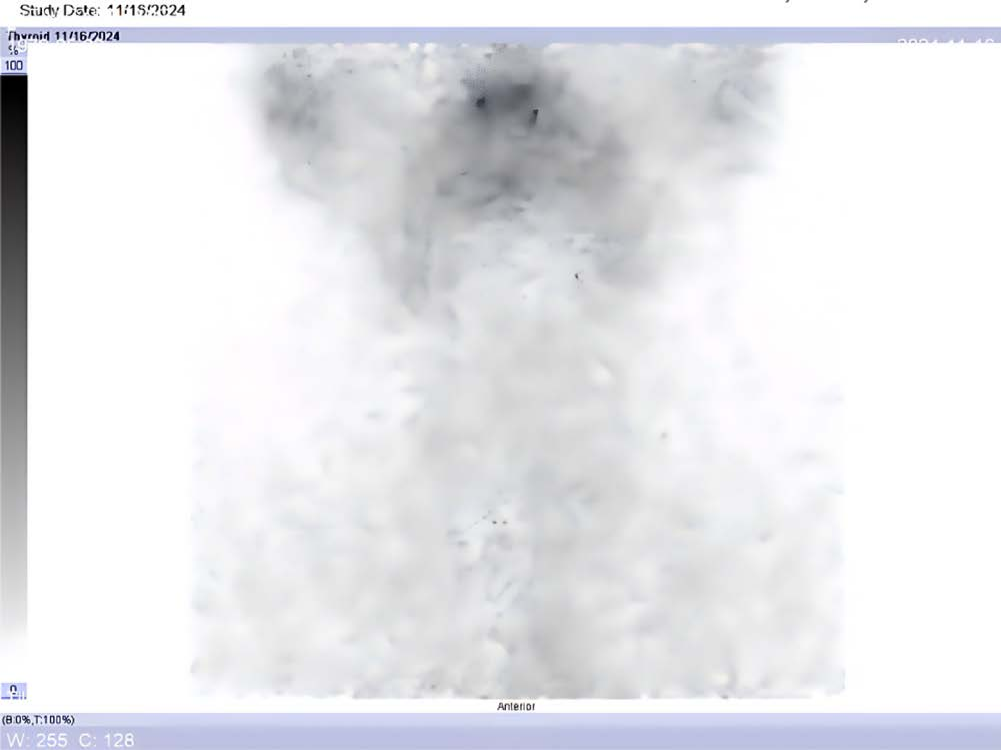
Pre-ablation ^99m^Tc-pertechnetate thyroid scintigraphy (anterior view) after total thyroidectomy shows no abnormal radiotracer uptake in the anterior neck, indicating no visible thyroid remnant in the thyroid bed.

On admission for radioiodine therapy (approximately 4 weeks post-surgery), the patient’s laboratory evaluation (in the hypothyroid, thyroid hormone withdrawal state) showed TSH 70.7 μIU/mL (normal 0.55–4.78), T3 0.39 nmol/L (normal 1.3–3.1), T4 28.0 nmol/L (66–181), FT3 2.36 pmol/L (3.5–6.5), FT4 3.14 pmol/L (11.5–22.7), consistent with post-thyroidectomy hypothyroidism. Importantly, the stimulated thyroglobulin (Tg) was 0.20 ng/mL (assay functional sensitivity < 0.1 ng/mL, reference < 55) and anti thyroglobulin antibody (TgAb) was 66.8 IU/mL (reference < 115), indicating essentially undetectable Tg with no interfering antibodies. These findings suggested no significant residual thyroid tissue or tumor at that time. A diagnostic thyroid bed scan with technetium-99m pertechnetate was performed prior to ablation (to assess for remnant tissue), which showed no focal uptake in the anterior neck (Fig. 1). This implied successful surgical removal of the thyroid with no visible residual tissue on scintigraphy, and proceeding with ^131^I ablation was deemed appropriate.

### 1.2. Timeline

**Figure 2.**
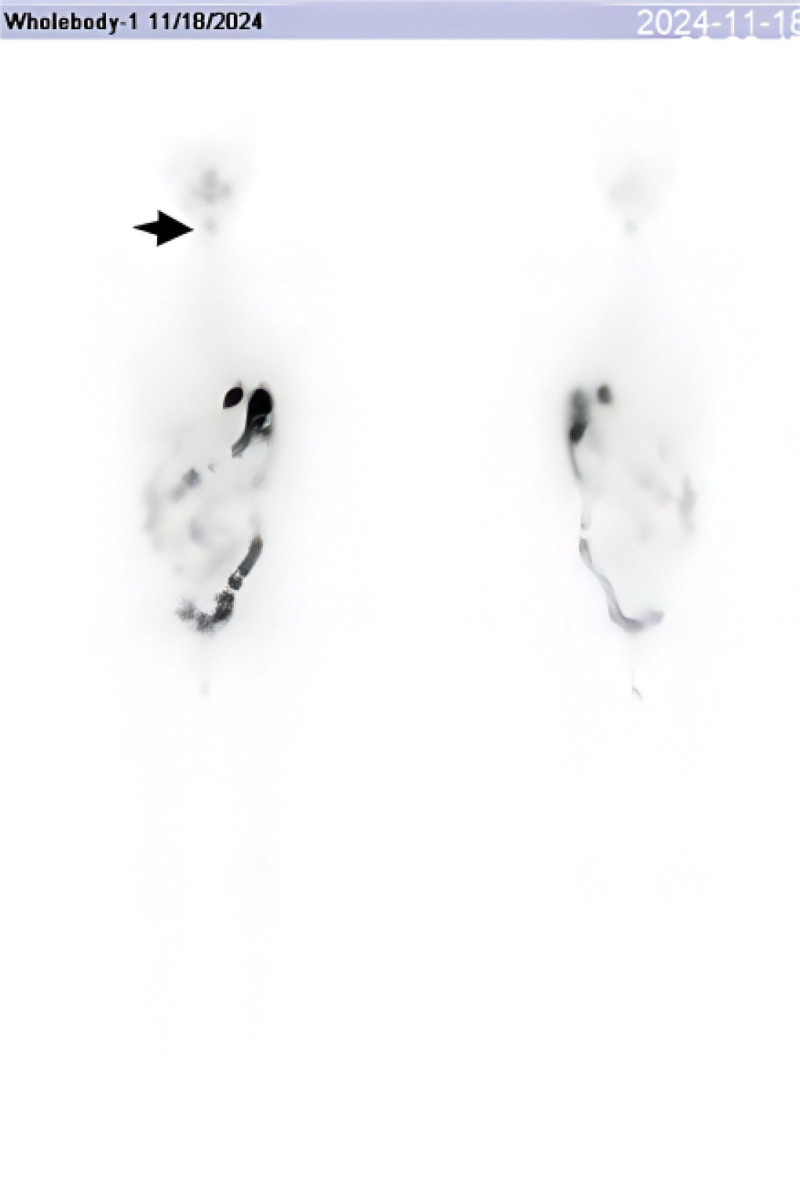
Post-therapy ^131^I whole-body scan obtained 48 hours after administration of 100 mCi shows a focal area of increased uptake in the midline upper neck corresponding to the thyroglossal duct region (arrow), with only minimal activity in the thyroid bed.

**Figure 3.**
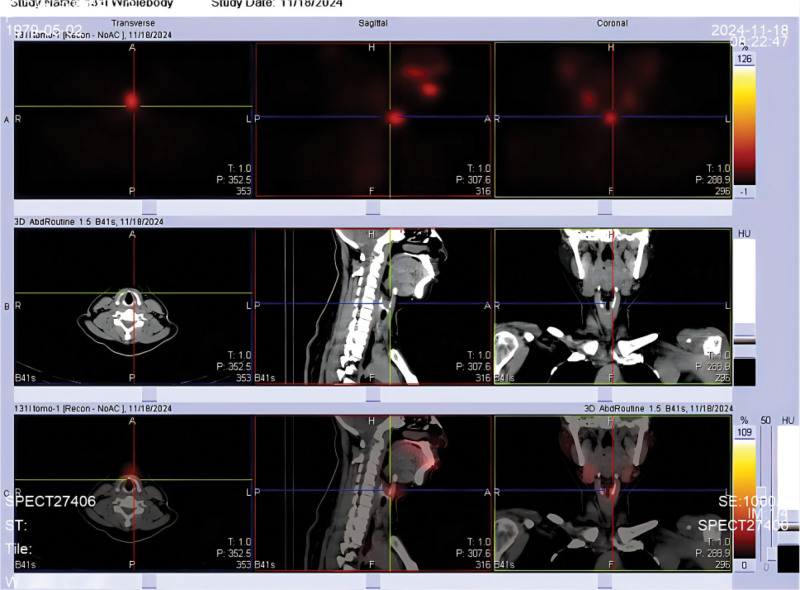
^131^I SPECT/CT fusion imaging at 48 hours localizes the uptake focus to the midline just inferior to the hyoid bone (crosshair), and the corresponding low-dose CT shows no discrete soft-tissue mass, consistent with a thyroglossal duct remnant. SPECT/CT = single photon emission computed tomography, computed tomography.

**Figure 4.**
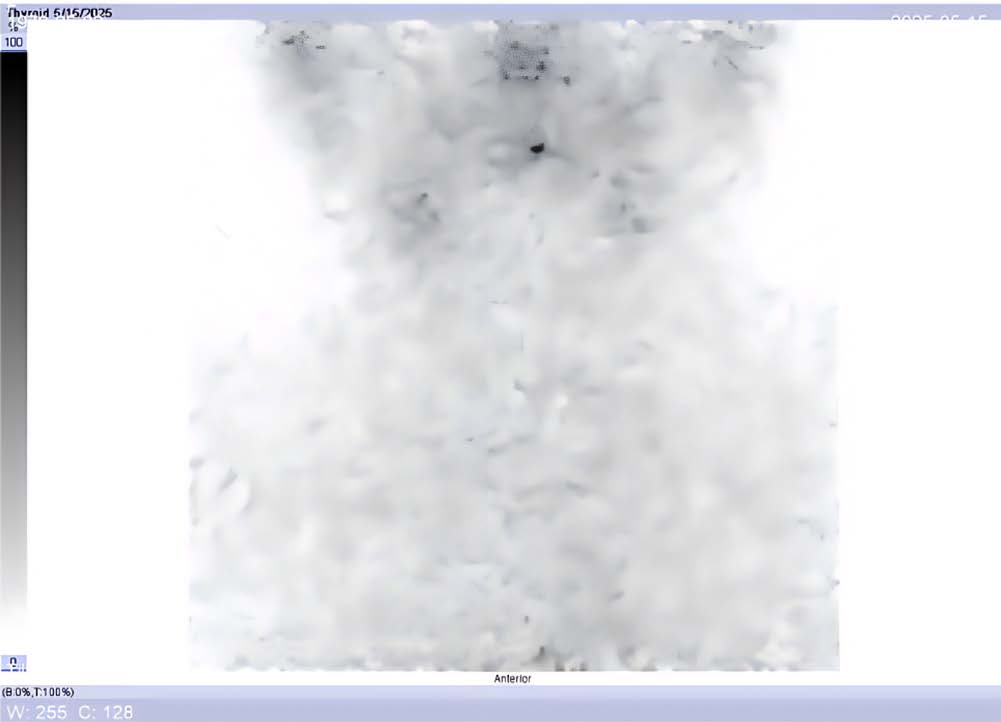
^99m^Tc-pertechnetate thyroid scintigraphy performed one month after levothyroxine withdrawal (endogenous TSH stimulation) shows no abnormal uptake in the anterior neck. TSH = thyroid stimulating hormone.

**Figure 5.**
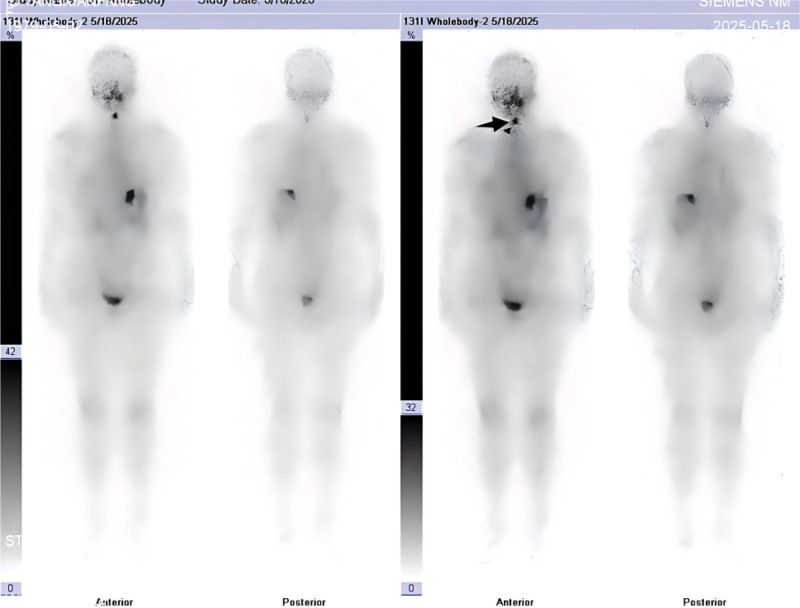
Twenty-four hour diagnostic ^131^I whole-body scan (3 mCi) performed 6 months after ablation demonstrates persistent focal uptake in the thyroglossal duct region above the midline of the neck (arrow), with no evident thyroid bed activity.

**Figure 6.**
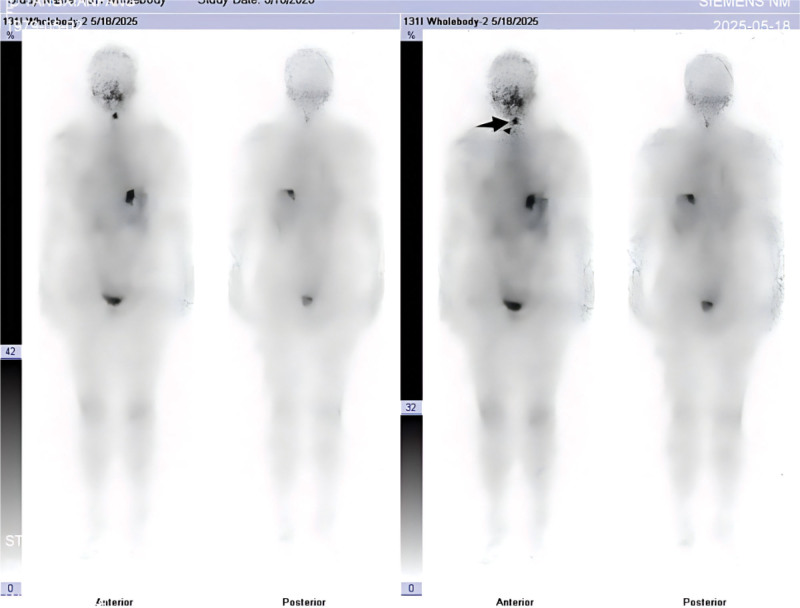
Forty-eight hour diagnostic ^131^I whole-body scan (3 mCi) shows persistent and slightly increased focal uptake in the thyroglossal duct region (arrow) compared with the 24-hour image, with no obvious uptake in the thyroid bed.

**Figure 7.**
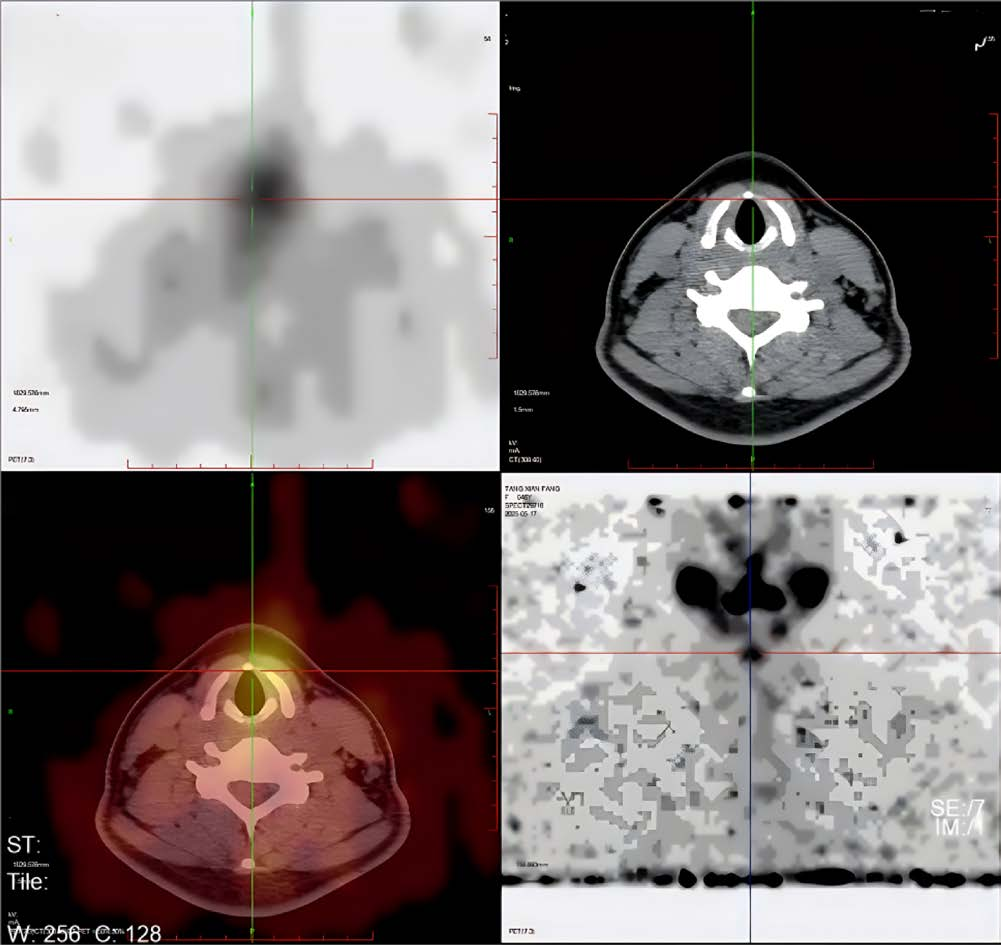
Fused ^131^I SPECT/CT at 48 hours demonstrates focal uptake anterior to the thyroid cartilage at the expected course of the thyroglossal duct (crosshair), with no corresponding soft-tissue lesion on CT, consistent with a thyroglossal duct remnant. SPECT/CT = single photon emission computed tomography, computed tomography.

1) Initial presentation (Month 0): incidental thyroid nodules detected on routine ultrasound. FNA confirms papillary thyroid carcinoma.
2) Month 1: total thyroidectomy and central compartment lymph node dissection (pathology: multifocal papillary carcinoma, N1a, stage I).
3) Month 2: preparation for ablation with thyroid hormone withdrawal. Laboratory: TSH > 70 μIU/mL; Tg 0.2 ng/mL (stimulated, negative TgAb). Pre-ablation ^99m^Tc thyroid scan shows no residual uptake (Fig. 1). 100 mCi ^131^I given for remnant ablation.
4) 48 hours post-ablation: ^131^I whole-body scan (WBS) shows focal uptake in midline upper neck (thyroglossal duct region), minimal uptake in thyroid bed (Fig. 2). Single photon emission computed tomography, computed tomography (SPECT/CT) localizes the focus to the thyroglossal tract, with no mass seen on CT (Fig. 3).
5) Month 3: initiated TSH suppression therapy with levothyroxine (100 μg daily).
6) Month 4 (1 month post-ablation follow-up): on levothyroxine – TSH 0.163 μIU/mL; Tg < 0.04 ng/mL; TgAb 44 IU/mL. No clinical or ultrasound evidence of disease.
7) Month 6 (4 months post-ablation): continued suppressive therapy – TSH 0.045 μIU/mL; Tg < 0.04 ng/mL; TgAb 15.9 IU/mL. As part of routine assessment, levothyroxine was then withdrawn for 1 month in preparation for a diagnostic radioiodine scan.
8) Month 7–8 (6 months post-ablation): off thyroid hormone for 4 weeks – TSH > 100 μIU/mL; Tg 0.20 ng/mL; TgAb 21.3 IU/mL (still undetectable Tg). thyroid static image (Fig. 4) , Low-dose (3 mCi) ^131^I administered for diagnostic scanning. 24-hour and 48-hour ^131^I scans again demonstrate persistent uptake in the thyroglossal duct region, no uptake in thyroid bed (Figs. 5 and 6). SPECT/CT confirms the same focal midline uptake with no structural lesion (Fig. 7).
9) Month 8+: levothyroxine resumed for long-term TSH suppression (target TSH 0.1–0.5 μIU/mL). Ongoing follow-up plan: clinical exam, neck ultrasound, and Tg monitoring every 6 to 12 months. No additional intervention pursued given stable imaging and undetectable Tg.

### 1.3. Diagnostic assessment and differential diagnosis

Post-ablation imaging revealed an unexpected finding: a focus of radioiodine uptake in the midline upper neck (above the thyroidectomy bed) consistent with the thyroglossal duct region. On the initial post-therapy scan (48 hours after 100 mCi ^131^I), a small but distinct focus of uptake was noted just above the thyroid bed in the anterior midline neck (Fig. 2). There was no appreciable uptake in the thyroid bed itself, indicating a successful ablation of thyroid remnant. A SPECT/CT was obtained immediately, which precisely localized the uptake to an area just inferior to the hyoid bone in the anterior midline (Fig. 3). The low-dose CT images showed no corresponding soft-tissue mass or cyst at that location. This anatomical region corresponds to the tract of the thyroglossal duct, suggesting the presence of functional thyroid tissue likely left behind from embryologic development (an ectopic thyroid remnant). Given the negative Tg, this uptake raised a diagnostic dilemma: Was this a true malignant focus, or a benign remnant? We undertook a thorough differential diagnosis analysis to avoid misinterpretation.

Several potential causes of ^131^I uptake in the midline neck were considered:

1) Radioiodine contamination or pooling: external contamination (for example, sweat or saliva containing ^131^I) on the skin or in the oropharynx can produce a false positive hotspot. We reviewed the imaging for signs of contamination (such as streaky uptake) and reexamined the patient. No removable contamination was identified. The focus persisted on delayed imaging 48 hours later and even on the follow-up diagnostic scans 6 months afterward, making transient surface contamination or secretions unlikely. Additionally, the patient’s neck was cleaned and re-imaged, with no change in the uptake focus, arguing against contamination or physiological retention (such as radioiodine retained in a cyst or sinus tract). Cases in the literature have highlighted unusual false positives from contamination (e.g., a patient’s face mask during COVID-19 causing a spurious uptake), but in this case the persistent and reproducible nature of the uptake and its fixed anatomical location pointed to an internal source.
2) Inflammation or infection: inflammatory lesions (such as an infected thyroglossal duct cyst or thyroiditis) can sometimes concentrate radioiodine due to increased vascular permeability or sodium iodide symporter expression in inflammatory cells. However, iodine uptake from inflammation is typically transient and would be expected to resolve over time. Our patient’s uptake focus remained essentially unchanged over a 6-month interval, arguing against a transient inflammatory cause. Moreover, she had no signs of infection or inflammation (no pain, swelling, or elevated inflammatory markers), and CT did not show any abscess or inflamed cyst.
3) Midline lymph node or metastatic tissue: a central compartment lymph node (such as the Delphian node near the thyroglossal tract) containing metastatic thyroid cancer could appear as midline uptake on a WBS. This was a critical consideration, given the patient’s known nodal metastases at initial surgery. However, ultrasound and the SPECT/CT images showed no discernible lymph node or tumor mass in the region of uptake. If a metastasis were present without a mass, one might expect rising Tg levels over time. In this patient, Tg remained undetectable on sensitive assays both on and off thyroid hormone, which is strongly against there being an ^131^I–avid metastatic deposit. Furthermore, there was no uptake in the thyroid bed or other distant sites on the scans, making an isolated single metastasis in the thyroglossal area less likely (though not impossible, as rare cases of thyroglossal duct cyst carcinoma or Delphian node metastasis have been documented). We also considered the possibility of occult residual thyroid tissue left in the neck (e.g., thyroid rest or pyramidal remnant), but again the lack of any uptake in the thyroid bed and negative ultrasound made this unlikely.
4) Benign ectopic thyroid tissue (thyroglossal duct remnant [TGDR]): the leading diagnosis was that this focus represented functional ectopic thyroid tissue within a TGDR. During embryologic development, the thyroid gland originates at the foramen cecum at the tongue base and descends to the neck, leaving a tract (thyroglossal duct) which normally involute. Remnants of thyroid tissue can persist anywhere along this tract (e.g., at the base of tongue – lingual thyroid, or near the hyoid bone). Such ectopic thyroid tissue can take up and organify radioiodine and produce Tg just like normal thyroid tissue. After total thyroidectomy, elevated endogenous TSH (or rhTSH stimulation) can stimulate these remnants, resulting in radioiodine uptake on a scan. In our patient, the location of the uptake on SPECT/CT – in the midline, just below the hyoid – is classic for a TGDR. There was no evidence that this tissue was malignant (no mass and no Tg production). Therefore, we concluded that the most likely explanation for the persistent uptake was an ectopic thyroglossal duct thyroid remnant that was not ablated by the initial radioiodine dose. This diagnosis is supported by multiple reports in the literature where TGDRs were visualized on post-ablation scans yet did not indicate recurrent cancer.

In summary, dynamic follow-up and multimodality imaging effectively excluded metastasis and other false positive causes, and the consistent findings pointed toward a benign TGDR as the cause of the ^131^I uptake. Recognition of this etiology was critical to prevent unnecessary aggressive interventions.

### 1.4. Intervention and follow-up

The patient’s management course was adjusted in light of the above findings. Initially, after the post-ablation scan demonstrated the thyroglossal region uptake, the nuclear medicine team advised a conservative approach given the undetectable Tg and the SPECT/CT localization suggesting benign tissue. The patient was started on levothyroxine suppression therapy one week after ablation to reduce TSH stimulation of any remaining thyroid cells. She was prescribed 100 μg daily, and her TSH was brought down to well below 0.1 μIU/mL within one month (Table 1). At the 1 month follow-up, the patient was clinically well; her TSH was 0.163 μIU/mL, and Tg remained < 0.04 ng/mL on suppressive therapy, indicating no biochemical evidence of disease. Neck ultrasound at that visit found only postsurgical changes and no masses in the thyroid bed or midline neck.

**Table 1:** Serial thyroid function and tumor marker results.

Time point (relative to surgery)	TSH (μIU/mL)	Tg (ng/mL)	TgAb (IU/mL)	T3 (nmol/L)	T4 (nmol/L)	FT3 (pmol/L)	FT4 (pmol/L)	Thyroid Hormone Status
1 mo post-surgery (pre-ablation; off levothyroxine, endogenous TSH stimulation)	70.700	0.20	66.8	0.39	28.00	2.36	3.14	Hypothyroid (withdrawal)
~1 mo post-ablation (on levothyroxine, suppressive therapy)	0.163	<0.04	44	–	–	8.66	23.46	TSH suppressed (over-replaced)
~4 mo post-ablation (on levothyroxine, suppressive therapy)	0.045	<0.04	15.9	–	–	5.25	18.20	TSH strongly suppressed
~6 mo post-ablation (diagnostic scan; off levothyroxine 4 wk, endogenous stimulation)	>100	0.20	21.3	–	21.00	0.80	1.71	Hypothyroid (withdrawal for scan)

All tests performed at the same laboratory; reference ranges: TSH 0.55 to 4.78 μIU/mL, Tg < 55 ng/mL, TgAb < 115 IU/mL, Total T3 1.3–3.1 nmol/L, Total T4 66–181 nmol/L, FT3 3.5–6.5 pmol/L, FT4 11.5–22.7 pmol/L.

Tg = thyroglobulin, TgAb = anti thyroglobulin antibody, FT3 = free triiodothyronine, FT4 = free thyroxine, TSH = thyroid stimulating hormone.

Over the next few months, the patient continued TSH-suppressive therapy. By about 4 months post-ablation, her TSH was further suppressed to 0.045 μIU/mL (free T4 was slightly above normal, indicating a high but tolerable dose of levothyroxine). Tg stayed undetectable (<0.04 ng/mL), and TgAb levels fell into the normal range (15.9 IU/mL). The patient reported good adherence to medication and had no signs of hypothyroidism or hyperthyroidism aside from mild subclinical hyperthyroid labs as intended.

At approximately 6 months after the initial ablation, a decision was made to perform a diagnostic ^131^I WBS for confirmation, given the earlier unusual finding. The patient stopped levothyroxine for 4 weeks to raise TSH (which rose to >100 μIU/mL), while maintaining a low-iodine diet. After endogenous stimulation, 3 mCi of ^131^I was administered orally, with scans obtained at 24 and 48 hours. The results mirrored the initial post-therapy scan: persistent focal uptake in the thyroglossal duct region and no uptake elsewhere (Figs. 5 and 6). In fact, the 48-hour image showed even more intense uptake in that focus (likely due to delayed imaging allowing more time for iodine trapping), confirming the presence of functioning thyroid tissue in that location. Once again, fused SPECT/CT at 48 hours showed the focus just anterior to the laryngeal area (level of thyroid cartilage) with no abnormalities on CT (Fig. 7). Concurrent labs at that time (on stimulation) showed Tg 0.20 ng/mL (unchanged from baseline and still in the undetectable range) and no TgAb interference. These findings provided additional evidence that this was a stable benign remnant, not a growing tumor.

Given these reassuring follow-up findings, the multidisciplinary team (including endocrinology, nuclear medicine, and surgery consultants) agreed on continued non-interventional management. The patient was restarted on levothyroxine promptly after the diagnostic scan, targeting a mildly suppressed TSH. We planned regular follow-up every 6 to 12 months with physical exams, neck ultrasound (especially to reevaluate the thyroglossal area for any emergent nodule or cyst change), and serum Tg/TgAb measurement. The TSH suppression goal was set to approximately 0.1 to 0.5 μIU/mL, in line with international guidelines for intermediate-risk DTC patients who are free of disease. This degree of suppression aims to reduce any TSH-driven growth of residual thyroid cells while minimizing risks of excessive thyroid hormone replacement. The patient was instructed on the importance of medication adherence and informed about signs of hyperthyroidism to watch for. No additional radioiodine therapy was given, as the clinical consensus was that the remnant was small and not likely to cause harm, and additional high-dose RAI would carry more risk than benefit in this context.

For trigger events that would prompt re-intervention, we advised that any significant rise in Tg on follow-up (e.g., consistently detectable or increasing Tg values) or new structural findings (a mass in the thyroid bed or along the thyroglossal tract on imaging) would warrant reevaluation. At that point, options would include surgical excision of the remnant or another dose of ^131^I, but only if clear evidence of disease appears. We emphasized a patient-tailored approach, aiming to avoid overtreatment while ensuring oncologic safety. To date, the patient remains well, with no symptoms and no evidence of disease progression at her most recent follow-up. She has expressed understanding and relief that no further invasive treatments were immediately needed, and she remains compliant with her suppressive therapy and follow-up plan.

### 1.5. Outcomes and patient perspective

At the 6-month mark after initial therapy, the patient had achieved excellent biochemical and structural response to treatment, aside from the isolated thyroglossal uptake which has been attributed to benign tissue. Her serum Tg remained undetectable throughout follow-up, and no new lesions were detected on ultrasound or SPECT/CT. The patient did not experience any symptoms of recurrence (such as neck swelling, dysphagia, or voice changes). Under TSH suppression, she reported mild symptoms of subclinical hyperthyroidism (occasional palpitations and heat intolerance), but these were tolerable and managed by adjusting the levothyroxine dose to keep free thyroid hormone levels just slightly above normal. Importantly, the patient expressed relief and reassurance that the persistent scan finding did not represent cancer recurrence. In her perspective, understanding that the uptake was due to a benign remnant was empowering and helped her avoid unnecessary anxiety. She was pleased to avoid an unnecessary second dose of radioiodine or surgery, and she trusted the plan for active surveillance. The patient has been educated on adherence to medication and follow-up tests. She remains motivated to continue follow-up, stating that the collaborative decision-making and clear explanations of the imaging findings have improved her confidence in the treatment plan. At last contact, she continues to do well on suppressive therapy, and no evidence of disease has emerged.

## 2. Discussion

The distinctive feature of this case is the persistent I-131 uptake in the same thyroglossal duct region after total thyroidectomy and I-131 ablation, with both serum Tg and TgAb remaining negative, and no resolution during 6 months of follow-up – a classic “functional imaging dissociation” phenomenon. There is currently no clear diagnostic or management pathway for this special situation in international guidelines, and significant controversy exists in clinical practice.

### 2.1. Potential causes of I-131 uptake in the thyroglossal duct region on planar imaging

Ectopic thyroid tissue: during embryonic development, the thyroid gland descends from the foramen cecum at the tongue base along the midline, possibly leaving remnants of thyroid tissue in the thyroglossal duct (e.g., near the hyoid bone or tongue base, referred to as TGDR or lingual thyroid).^[1]^ These ectopic tissues can show uptake and organification of iodine as well as production of Tg, functioning similarly to normal thyroid tissue.^[1]^ After total thyroidectomy, the elevated TSH stimulates these previously dormant ectopic tissues, resulting in local accumulation on I-131 scans.^[2]^

Metastatic thyroid carcinoma: although uptake in the thyroglossal duct region is most often caused by benign ectopic thyroid remnants, metastatic DTC must also be considered. If the primary cancer involves the TGDR (e.g., papillary carcinoma in a thyroglossal duct cyst), the site itself can become a malignant I-131-avid lesion. Central neck lymph node metastasis, such as a Delphian node near the hyoid, may also appear as a hotspot in the midline on imaging. Generally, any abnormal uptake outside the thyroid bed on postoperative ^131^I WBS should first raise suspicion of metastatic or residual tumor, but additional examination is required for differentiation, as ectopic thyroid tissue can be indistinguishable from metastasis on imaging.^[1]^

Inflammation or granuloma: radioiodine may also transiently accumulate in inflammatory lesions due to increased local blood flow and capillary permeability.^[1]^ For example, infection, cystic inflammation, or granulation tissue in the thyroglossal duct area may result in mild uptake, but such uptake generally does not persist and subsides as inflammation resolves. I-131 can also accumulate abnormally in head and neck inflammations such as sialadenitis, sinusitis, or surgical wounds.^[1]^ However, persistent uptake over 6 months as seen in this case makes inflammation less likely.

Other factors (retention/secretion of ^131^I and contamination) can also generate spurious hotspots. False-positive anterior neck uptake has been documented from retention of secretions around airway devices and from contamination by ^131^I – containing nasal/oral secretions (e.g., saliva on masks or skin), which can be resolved by removing the source, skin cleansing, repositioning, or delayed rescanning.^[2]^ Such contamination usually remains on the skin surface and can be distinguished by repositioning, delayed scanning, or skin cleaning.^[1]^

### 2.2. Detection rate of thyroglossal duct remnants on postoperative I-131 imaging

It is reported that approximately 30% to 35% of DTC patients undergoing diagnostic I-131 scans under TSH stimulation after total thyroidectomy show abnormal uptake along the thyroglossal duct in the anterior midline.^[3]^ With higher-sensitivity I-131 SPECT/CT, the detection rate can reach nearly 50%.^[4]^ This suggests that occult TGDRs are not uncommon, challenging the traditional notion that ectopic thyroid tissue is rare.^[3]^ Thyroid tissue can persist anywhere along the migration pathway from the tongue base to the retrosternal area.^[3]^ The incidence of ectopic thyroid in the general population is approximately 1/1,00,000 to 3,00,000, but among patients with thyroid disease, it increases to 1/4000–8000.^[3]^ Autopsy reports indicate that the rate of ectopic thyroid tissue can reach 10% to 40%.^[3]^ Ectopic thyroid is more common in females (especially in Asian populations), with about 90% located at the tongue base (lingual thyroid), and the rest distributed along the midline from sublingual to pretracheal areas.^[3]^ Therefore, the appearance of small TGDRs on I-131 imaging under elevated TSH after surgery is not uncommon, even if there were no midline masses preoperatively.^[3]^

### 2.3. *Can* ^*99m*^*Tc thyroid scintigraphy detect thyroglossal duct remnants?*

Conventional ^99m^Tc thyroid scintigraphy can depict ectopic (including lingual) thyroid tissue, and hybrid SPECT/CT further refines anatomic localization and characterization, as shown in recent reports.^[5]^ Nonetheless, its sensitivity for tiny TGDRs remains limited because of planar resolution and partial-volume effects. In our case, the TGDR was not detected by ^99m^Tc scintigraphy, most likely because of these technical constraints, rather than an intrinsic inability of the radiotracer itself.

Conventional ^99m^Tc thyroid scintigraphy is often unable to detect small remnants in the thyroglossal duct, as seen in this case. This is because ectopic thyroid tissue in the duct is usually of low function and small volume, and when normal thyroid tissue exists or during TSH suppression, uptake is “silent.”^[3]^ Studies have found that in patients post-I-131 ablation, only 0.9% showed hyoid region uptake on diagnostic scans under TSH suppression, while the detection rate significantly increased under TSH stimulation.^[3]^ In postoperative DTC, ^99m^Tc-MIBI SPECT/CT shows limited sensitivity for thyroid remnants compared with ^131^I WBS (68% vs 100%), although it may better depict nodal disease in selected cases.^[6]^ Similarly, traditional planar I-131 imaging can miss TGDRs due to low resolution and lack of anatomical correlation.^[3]^ Therefore, advanced imaging techniques (such as ^123^I imaging or SPECT/CT fusion) are recommended to improve detection.^[3]^

### 2.4. What are the advantages of SPECT/CT for diagnosing thyroglossal duct remnants?

Planar I-131 imaging is limited in anatomical localization, especially for small midline foci like TGDRs. SPECT/CT fusion imaging is valuable in this setting:

Precise localization: SPECT/CT combines functional and anatomical images, enabling precise identification of hotspots along the thyroglossal duct path.^[4]^ Typical TGDR appears as a midline hotspot several millimeters to 1 cm above the thyroid bed.^[3]^ It may present as an oval focal uptake (suggesting a small nodule) or as a linear extension along the duct (suggesting a string-like remnant).^[3]^ SPECT/CT-detected uptake along the thyroglossal duct pathway without evidence of metastasis can be diagnosed as TGDR.^[4]^

Differentiating benign/malignant and false positives: SPECT/CT provides CT details that help determine if the focus corresponds to a solid tissue nodule or only to air/mucus, indicating false positive.^[2]^ SPECT/CT is the most effective tool for distinguishing between physiological/false positives and true lesions.^[1]^ In summary, SPECT/CT greatly enhances the specificity of I-131 imaging and avoids misdiagnosing benign uptake as tumor metastasis.^[4]^

### 2.5. Other imaging modalities for thyroglossal duct assessment

Besides SPECT/CT, high-frequency ultrasound of the neck midline can detect small nodules or cysts, especially near the hyoid, while magnetic resonance imaging/CT can visualize deeper or tongue base lesions. ^18^F-FDG positron emission tomography, computed tomography is generally used in cases with elevated Tg but negative I-131 scans; it can help differentiate inflammation from tumor when necessary. In this case, I-131 SPECT/CT is the preferred imaging modality for thyroglossal duct hotspots.^[4]^

### 2.6. *Can thyroglossal duct remnants be ablated by I*-*131?*

TGDRs are functional thyroid tissue and generally can concentrate I-131 and be destroyed by ablation. Most such foci shrink or disappear after the first ablation.^[4]^ One study reported a higher I-131 ablation success rate in patients with TGDR (79% vs 41%, *P* < .01) compared to those without.^[3]^ However, about 17% of TGDR foci may persist after initial treatment, showing “resistance” or incomplete clearance.^[4]^ The reasons include small size, low-iodine uptake, and poor blood supply, which limit the effective I-131 dose received by the remnant. Some suggest sodium iodide symporter expression may be lower in ectopic tissue, reducing iodine uptake efficiency.^[3]^ Therefore, some TGDRs may require repeated ablation or long-term follow-up.

### 2.7. Does TGDR affect interpretation of serum Tg and TgAb?

TGDR, as thyroid tissue, can secrete Tg under TSH stimulation. Thus, even after total thyroidectomy, the presence of minimal ectopic remnant may cause poststimulation Tg to be nonzero.^[4]^ Studies show that stimulated Tg is significantly higher in patients with TGDR.^[4]^ Conversely, if TGDR exists but poststimulation Tg remains undetectable, one should consider interference (e.g., by TgAb) or tissue inactivity. Literature reports that 23% of patients with remnant uptake but undetectable Tg after I-131 ablation had interference from TgAb or heterophile antibodies^[7]^; after correcting for interference, some had traceable Tg.^[7]^ Even so, about 19% of patients had undetectable Tg by all methods, likely due to minimal tissue volume and suppressed function by levothyroxine.^[7]^ TGDR is often seen in patients without autoimmunity (as in this case), but persistent ectopic tissue in those with thyroiditis may stimulate TgAb production, further affecting Tg measurement. Thus, both imaging and laboratory results should be integrated to avoid unnecessary intervention based solely on Tg.^[7]^

### 2.8. Impact of significant Tg decline, negative TgAb, but persistent imaging positivity in this case: functional remnant vs false positive?

Six months after surgery, the patient’s Tg had declined significantly, TgAb was negative, but I-131 uptake in the thyroglossal duct persisted—such discordance requires careful interpretation:

Functional remnant: declining Tg suggests most thyroid tissue (especially tumor) has been cleared, but does not guarantee the absence of all functional tissue. Small benign remnants or ectopic tissue may be too small to elevate Tg, but may still concentrate I-131 under high TSH.^[8]^ In particular, TGDRs with a few functional cells may be visible on imaging but contribute little to serum Tg. Studies show that patients with TGDR uptake post-ablation often have minimal thyroid bed remnant and only tiny ectopic tissue.^[3]^ Such patients typically respond well to I-131, with little risk of widespread tumor.^[3]^

False positive: conversely, a hotspot may result from nonspecific causes (e.g., contamination, retained secretions), and the patient may have no functional remnant, explaining low/stable Tg.^[2]^ False positive: conversely, some hotspots arise from nonspecific causes (e.g., contamination or urinary/secretory retention), in which case Tg typically remains low or undetectable despite apparent lesions on ^131^I WBS. This pattern has been repeatedly described in recent literature, including renal/urinary-tract–related uptake and post-therapy false positives that resolve after source removal or delayed rescanning.^[9]^ There are reports of patients with repeated lung foci on scan and persistently low Tg, later confirmed to be false positives.^[8]^ Thus, persistently low Tg supports the absence of significant viable thyroid cancer, and imaging positivity should be considered for false positive causes.

### 2.9. Is repeat I-131 ablation needed?

Guidelines such as the 2015 American Thyroid Association recommend against routine overtreatment with I-131 in low-risk DTC patients.^[10]^ In patients with only minimal benign tissue remaining and undetectable Tg after initial ablation, further high-activity iodine treatment is more likely to cause harm than benefit. A prospective cohort study randomized 94 patients with residual uptake but negative Tg after first ablation into observation versus re-treatment. The rates of new abnormal findings (Tg rise or imaging lesion) did not differ significantly between groups (about 17% vs 21%, *P* > .6), and about one-third in the observation group had spontaneous resolution.^[11]^ Thus, second I-131 ablation did not improve outcomes over observation.^[11]^ European and American guidelines also favor individualized management; for low-risk patients without structural disease, close monitoring is preferred over immediate repeat ablation.^[10]^ Before conservative management, ensure the uptake focus is truly benign (SPECT/CT in the duct tract with no anatomical abnormality, negative TgAb, and negative neck ultrasound). TSH suppression and regular follow-up are recommended, with further intervention only if there is functional or structural progression. Avoiding unnecessary ablation reflects the principle of risk stratification and overtreatment avoidance,^[10]^ in line with American Thyroid Association and European Thyroid Association recommendations. This case demonstrates that in patients with negative Tg and isolated TGDR uptake post-ablation, clinical judgment should take precedence over empirical re-treatment.^[3]^

### 2.10. Management recommendations and follow-up strategies for persistent I-131 uptake

For persistent uptake in the thyroglossal duct after I-131 ablation, clinicians should weigh the risks and need for invasive intervention:

Further evaluation: SPECT/CT or ultrasound to confirm the nature of the focus – true remnant/tumor or false positive. Benign ectopic remnant is likely if no suspicious findings or Tg elevation.

Conservative observation: for confirmed/suspected benign ectopic tissue, observation is appropriate.^[12]^ TGDR is not rare and usually asymptomatic,^[13]^ with good prognosis if only small residual tissue remains after I-131.^[3]^ Regular Tg (every 6–12 months) and ultrasound are recommended; if stable, observation continues. Many foci may atrophy or lose function over time.

Additional I-131 therapy: in cases with persistent significant uptake after first ablation and higher risk stratification (e.g., intermediate/high risk, detectable Tg), a second ablation may be considered after weighing the risks. Preparation with TSH stimulation and low-iodine diet is necessary.^[4]^

Surgical intervention: reserved for highly suspicious foci (growing nodule, cytologically confirmed malignancy) or symptomatic patients. Sistrunk procedure is standard for cystic lesions; more extensive surgery is needed for lesions at the tongue base or with lymph node metastasis. Surgery is not routine for small benign foci unless symptomatic or at patient request.^[14]^

Long-term follow-up: all patients should have ongoing follow-up with Tg/TgAb every 6 to 12 months and regular neck imaging. Repeat diagnostic scans or SPECT/CT can be considered after 1 to 2 years. If Tg remains undetectable and imaging negative for years, follow-up can be extended. Any Tg rise or new symptoms should prompt comprehensive reassessment (including I-131 imaging, ^18^F-FDG positron emission tomography, computed tomography, or magnetic resonance imaging/CT as needed).

This case demonstrates that after successful ablation and with negative Tg/TgAb, persistent thyroglossal duct region uptake most often represents benign ectopic thyroid tissue rather than residual or recurrent tumor. SPECT/CT and multimodal imaging are crucial for accurate localization and management, supporting individualized follow-up and avoidance of overtreatment. Dynamic observation of such phenomena may help refine the global consensus for postoperative DTC management.

### 2.11. Clinical implications

Persistent I-131 uptake in the thyroglossal duct region may indicate tumor, but is often benign ectopic tissue. This case illustrates that TGDRs may be insensitive to I-131 therapy. For patients with negative Tg/TgAb and isolated persistent uptake, emphasis should be placed on multimodal imaging (e.g., SPECT/CT), combined biochemical and imaging assessment, individualized follow-up, and avoidance of overtreatment. At the same time, vigilant monitoring and timely intervention ensure adequate tumor control. This case highlights the challenge of “functional imaging discordance” in DTC follow-up and offers a reference for personalized management. Multicenter collaborative studies are recommended to further clarify the clinical significance of persistent thyroglossal duct uptake.

From a diagnostic perspective, several differential diagnoses were systematically considered in this patient. First, contamination of the skin or retention of saliva or nasal secretions containing ^131^I was unlikely, because the focus did not change after careful skin cleansing, repositioning, and delayed imaging, and the hotspot remained fixed in the same anterior midline position on all scans.^[1,2,8,9,12]^ Second, a midline lymph node or inflammatory lesion was excluded, since high-resolution ultrasound and SPECT/CT showed no solid nodule or inflammatory mass along the thyroglossal tract region.^[3,4,6,13,14]^ Third, residual thyroid bed tissue or metastatic disease was considered improbable, given the repeatedly negative thyroid bed on WBS and SPECT/CT and the persistently undetectable or very low Tg values.^[3,4,7]^ Taken together, these findings strongly support a benign functional TGDR as the most likely explanation for the persistent ^131^I uptake.^[3,4,13]^

The management strategy in this case was therefore deliberately conservative. In accordance with current risk-adapted recommendations for low- and intermediate-risk DTC, we targeted partial TSH suppression with levothyroxine, aiming for a TSH concentration of approximately 0.1 to 0.5 μIU per mL, while avoiding overt hyperthyroidism.^[10,15]^ Follow-up was scheduled every 6 to 12 months with clinical examination, neck ultrasound, and measurement of serum Tg and TgAb. Escalation of therapy, such as repeat ^131^I administration or surgical exploration of the thyroglossal tract, was reserved for clearly defined triggers, including the appearance of a new solid nodule in the thyroid bed or along the tract, a sustained rise in Tg, or structural progression on imaging.^[7,10,11,15]^

This report has several limitations. It describes a single patient, and there was no histologic confirmation of the TGDR, because surgery was not clinically indicated. Nevertheless, the very consistent functional and anatomical imaging findings together with the excellent biochemical response make a benign interpretation highly probable. From a practical point of view, this case suggests a simple and generalizable decision algorithm for patients who show isolated midline ^131^I uptake after thyroid remnant ablation. If SPECT/CT confirms that the focus is located along the expected thyroglossal duct pathway, if ultrasound and CT do not demonstrate a corresponding solid lesion, and if Tg remains undetectable under appropriate TSH conditions, conservative management with TSH suppression and periodic imaging can usually be adopted.^[3,4,7,10–12,15]^ In contrast, if a discrete nodule is identified or Tg begins to rise, further intervention, including repeat ^131^I therapy or surgery, should be considered.^[7,10,11,15]^

In the broader context of thyroid and other endocrine related cancers, recent advances in radionuclide based nanotheranostic platforms for hepatocellular carcinoma and in Cerenkov radiation activated cancer theranostics demonstrate how radiopharmaceuticals can be integrated with multifunctional carriers and optical readouts to further refine individualized management strategies in the future.^[16-19]^

### 2.12. *Practical algorithm for incidental thyroglossal duct uptake after* ^*131*^*I therapy*

1. Confirm the finding with ^131^I SPECT/CT to localize the focus along the thyroglossal duct tract and exclude skin contamination.
2. Integrate stimulated and suppressed Tg/TgAb values together with high-resolution neck ultrasound to exclude thyroid bed remnant and nodal metastasis.
3. If imaging shows a tiny midline focus without a solid mass, Tg remains undetectable or very low, and there is no structural disease, classify the lesion as a likely benign TGDR and continue TSH suppression with clinical, biochemical, and ultrasound follow-up every 6 to 12 months.
4. Consider repeat high-dose ^131^I therapy or surgery only when there is structural progression, new solid nodules, or rising Tg suggestive of recurrent carcinoma.

## Author contributions

**Conceptualization:** Zu Guo Li, Lei Zhang, Bi yun Mo, Wei Fu.

**Data curation:** Jie Qin, Xing yu Mu, Wei Xia Chong.

**Formal analysis:** Yu Long Zeng, Xing yu Mu.

**Investigation:** Jing Ze Li.

**Methodology:** Jing Ze Li.

**Resources:** Ping Yu.

**Supervision:** Wei Fu.

**Writing – original draft:** Jie Qin.

**Writing – review & editing:** Jie Qin, Ping Yu.
